# Use of chick neural tube for optimizing the PSM and epithelial somites electroporation parameters: A detailed protocol

**DOI:** 10.14440/jbm.2018.253

**Published:** 2018-06-07

**Authors:** Muhammad Abu-Elmagd

**Affiliations:** Center of Excellence in Genomic Medicine Research, King Abdulaziz University, Jeddah, Kingdom of Saudi Arabia

**Keywords:** electroporation, neural tube, presegmented mesoderm, somites

## Abstract

Somite myogenesis is one of the crucial early embryonic events that lead to the formation of muscular tissue. A complex of dynamic gene regulatory networks masters this event. To understand and analyze these networks, there remains a genuine need for the use of a reproducible and highly efficient gene transfer technique. *In vivo* electroporation has proven to be amongst the best approaches in achieving a high level of gene transfer. However, unoptimized electroporation conditions can directly cause varying degrees of cellular damage which may induce abnormal embryonic development as well as changes in the endogenous gene expression. Presegmented mesoderm and epithelial somites are not easy to electroporate. Chick neural tube has served in many functional studies as an ideal experimental model organ which is both robust and easily manipulated. In the current detailed protocol, the neural tube was used as a tool to optimize the electroporation conditions which were subsequently applied in the electroporation of the presegmented mesoderm and epithelial somites. The protocol highlights important notes and hints that enable reproducible results and could be applied in the *in vivo* electroporation of other chick embryo tissues.

## INTRODUCTION

Somite formation is a complex of developmentally orchestrated cell signaling that leads to skeletal muscle and vertebral column formation. Somite myogenesis participates in the formation of ribs, cartilage, tendons, ligaments and limb buds. This process begins by segmenting a solid rod of mesoderm, the presegmented mesoderm (PSM) from which a new epithelial somite (ES) is produced every 90 min. Once a somite is formed, it starts to differentiate from the caudal to the rostral direction to form dorsal (epaxial) and ventral (hypaxial) domains [1]. The dorsal domain gives rise to the dermis and skeletal muscles while the sclerotome migrates to give rise to the vertebrae, smooth muscle cells, fibrocytes, chondrocytes, osteocytes and endothelial cells [2].

A considerable number of studies investigating gene expression and the governing regulatory networks during somite myogenesis have been carried out (reviewed in [3]). However, these networks are yet to be completely understood [4]. Gain- and loss-of-gene expression using either *in vivo* and/or *in vitro* electroporation is a very powerful approach for manipulating gene function [5]. This includes electroporating single (aptamer) [6] or double [7] strand DNA, microRNA inhibitor (antagomir) [8], siRNA [9], morpholino (MO) [10], and Crispr-Cas9 [11] into the whole embryo, the neural tube (NT), neural crest, somites, retinal explants, and brain. However, gene transfer by electroporation has few main limitations which include direct damage to the cells, difficulty in controlling the number of electroporated cells, and possible off-target gene transfer. Some of these disadvantages could be controlled through a suitable optimization of the electroporation conditions [12].

Amongst animal model systems, chick embryo offers many advantages as a simple, experimental, and manipulative embryonic model for the study of different aspects of development. Advantages of the chick embryo include a high level of similarity with the human genome, and the low cost and ease by which it can be obtained and developed in the laboratory. *In vivo* chick embryo electroporation approach has been used to study gene function and regulation during somite myogenesis [13]. However, from our own experience and as has been reported by Wang and his colleagues, targeting muscle cells is not easy at any stage of development [14]. On the other hand, and due to its structural advantages, NT has served as a robust and easy to target embryonic tissue by electroporation [15]. Based on this, we aimed to present a detailed and enhanced *in vivo* chick PSM and ES electroporation protocol which can ensure efficiency and reproducibility.

In the first stage of the protocol, electroporation conditions were optimized by electroporating IRES-GFP expression construct into the NT of HH16 chick embryos. A number of electroporation conditions were tested after which the embryo survival rate, normal development and GFP expression were assessed. At the end of this stage, optimal electroporation conditions were determined. In the second stage, the parameters which resulted in efficient electroporation of the NT in stage 1 were applied to electroporate the PSM and ES after which the survival rate, normal embryonic development, and GFP or RFP expression intensity were critically assessed. Each experiment was repeated to ensure the reproducibility.

## MATERIALS

### Reagents

✓ Diethyl Pyrocarbonate (DEPC) (Sigma, cat. # 40718)
✓ EtOH (Sigma, cat. # 459836)
✓ Fast Green FCF (Sigma, cat. # F7252)
✓ Indian ink (Pelikan, cat. # 221143)
✓ Morpholino standard control oligo (5’-CCTCTTACCTCAGTTACAATTTATA-3’), Gene-Tools
✓ Paraformaldehyde (Sigma, cat. # 158127)
✓ PBS tablets (Sigma, cat. # P4417)
✓ pCMV-IRES-GFP (Addgene, plasmid # 78264)
✓ pCMV-IRES-RFP (Addgene, plasmid # 33337)
✓ Penicillin/Streptomycin 10000 units (Sigma, cat. # P4333)
✓ Qiagen EndoFree Plasmid Maxi kit (Qiagen, cat. # 12362)
✓ *In situ* hybridization (ISH) reagents: The detailed ISH protocol including reagents is publicly available from Geisha chicken embryo gene expression database [16].

### Equipment

✓ Chicken egg incubator with rotating shelves (Nanchang Panche Technology Co. Ltd., Model # BZ-176)
✓ BOD incubator (Saini Science Industries, model: SSI series)
✓ Stereo binocular microscope on post stand (World Precision Instruments, cat. # Z-LITE-186)

**HINT:** The dissecting microscope should have at least 20 cm working distance between the objective lens and the base of the microscope to allow easy handling of the chicken egg and embryo manipulation.

✓ High intensity fibre optic gooseneck illumination source (World
✓ Precision Instruments, cat. # PZMIII)
✓ Fluorescence stereomicroscope (Leica M205 FCA) equipped with AlexaFluor 488 ηm and 565 ηm filters
✓ Micro-torch, Piezo Butane gas burner (Gunz Dental, model GB-2001, cat. # KAM2001)
✓ Right-handed micromanipulator (Harvard Apparatus, cat. # EC164-0056)
✓ Left-handed micromanipulator (Harvard Apparatus, cat. # EC 1 60-0570)
✓ Mounting bases (Harvard Apparatus, cat. # EC 1 69-0225)
✓ Microinjector with microcapillary holder (Eppendorf FemtoJet 4i, cat. # 5252000013)

**NOTE:** FemtoJet 4i microinjector has the capability to build up an internal pressure and hence there is no need for an external pressure supply unit.

✓ Intracel TSS20 Ovodyne electroporator (Abbotsbury Engineering Ltd, cat. # 01-916-02)
✓ Intracel EP21 current amplifier (Abbotsbury Engineering Ltd, cat. # 01-918-02)
✓ Micropipette puller, Flaming/Brown (Harvard Apparatus, model P97)

**NOTE:** P97 is a horizontal microcapillary puller which has many advantages over the vertical puller models (such as KOPF, model 720). Amongst these is the capability to run a ramp test which determines the microcapillary melting temperature at which the glass can be pulled. Ramp test is very useful especially when either the P97 puller is used for the first time, there is a need to replace the filament of the puller, or a new type of microcapillaries is required for injection.

✓ Electrode holder (World Precision Instruments, model M3301EH)
✓ NanoDrop 2000/2000c spectrophotometer (Thermo Fisher, cat. # ND-2000)
✓ Tubes rocker (Thermo Scientific, cat. # 11-676-333)
✓ Centrifuge MiniSpin (Eppendorf, cat. # 5452000018)

### Miscellaneous dissection and injection tools

✓ Corning syringe filters, 0.2 μm (Sigma, cat. # CLS431212)
✓ Microcapillaries, Borosilicate thin wall with filament glass (Harvard Apparatus, cat. # 300035)
✓ Microloader tips (Eppendorf, cat. # 930001007)
✓ Pasteur pipettes (Sigma, cat. # Z331767)
✓ Platinum wire, 0.5 mm diameter (World Precision Instruments, cat. # PTP201)
✓ Tungsten wire, 0.125 mm diameter (World Precision Instruments, cat. # TGW0515)
✓ Watchmaker’s forceps, straight 11 cm, No. 5 (Pyramid Innovation Ltd., cat. # R35205-E)
✓ Watchmaker’s forceps, curved 12 cm, No. 7 (Pyramid Innovation Ltd., cat. # R35207-E)

### Recipes

#### Fast Green

Prepare in water at a final concentration of 1%, sterilize using 0.22 µm syringe filter, and store at 4°C. Mix Fast Green with the DNA to be electroporated at a ratio of 1:10 to aid in visualizing the injected DNA.

#### Indian ink buffer

Add 5 drops of Indian ink using 1 ml plastic Pasteur pipette to 15 ml 1 × PBS/Penicillin-Streptomycin (100 µg/ml), then filter sterilize using 0.22 µm syringe filter. This solution should be freshly prepared when required for each experiment.

#### Tungsten needles

Sharpen the Tungsten wire using a micro-torch flame then insert the sharpened-wire into a glass rod by slightly melting the glass using the flame. The Tungsten needles should be kept in a dissecting tools’ box to avoid damage to the needles’ fine tips. Tungsten needles are used for embryo microdissection and removal of the inner vitelline membrane.

#### Egg incubation

Incubate chicken eggs at 38°C and 75% humidity to obtain either stage HH12 or HH16 embryos according to Hamburger and Hamilton timetable [17].

**HINTS:** (1) Fertilized eggs should be pathogen free; (2) Upon arrival to the lab, store eggs in the BOD incubator at 15°C for 24 h prior incubation to allow the eggs to settle; (3) Eggs stored for more than one-week at 15°C should not be used for electroporation since they usually develop poor quality embryos; (4) Temperature and humidity of the eggs’ incubator should be monitored; and (5) Rotation of the eggs during incubation is strongly recommended (to prevent adhesion of the egg membranes) which usually leads to producing high quality embryos.

#### Electroporation station setting up

**Figure 1.**
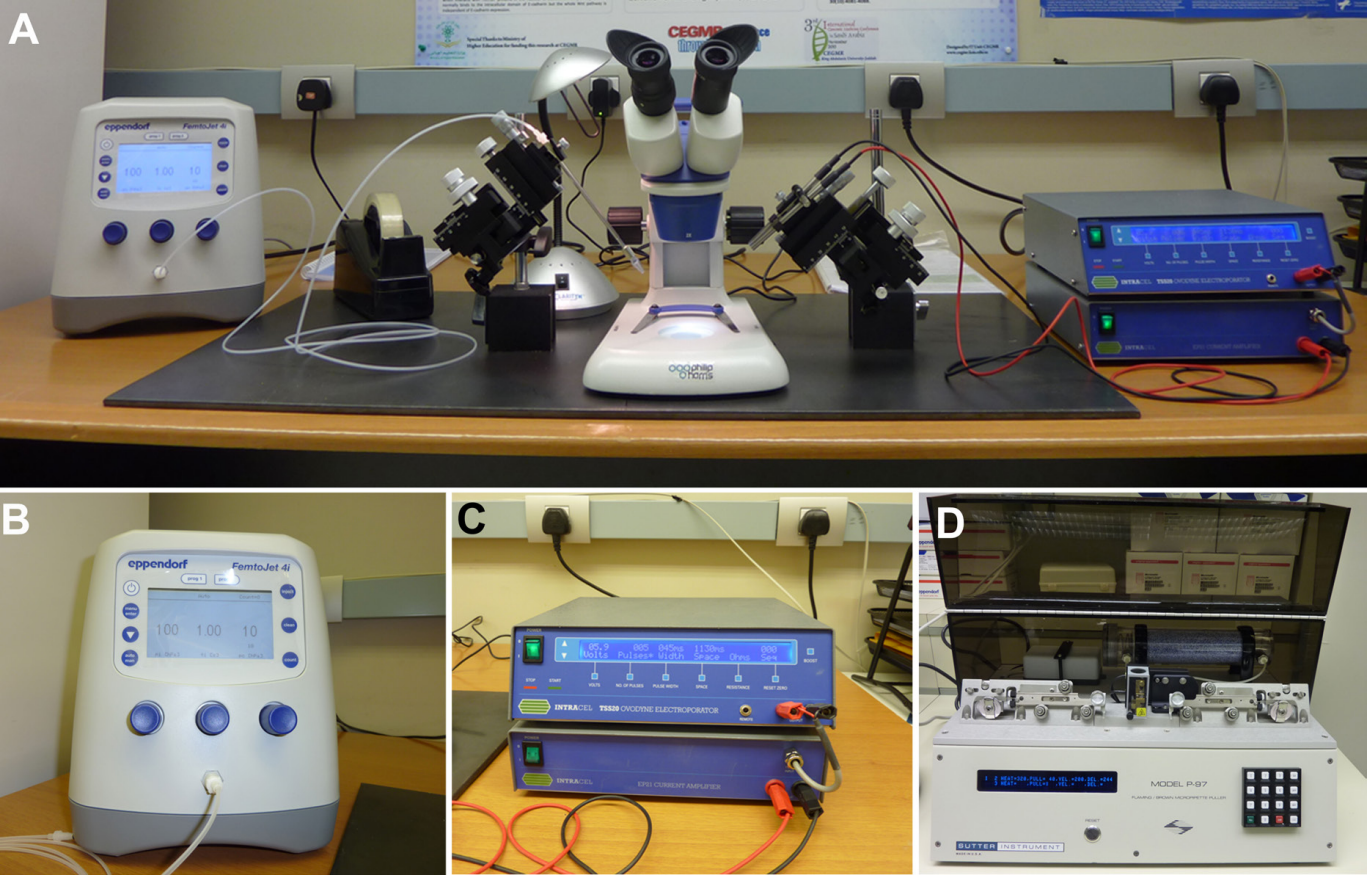
Electroporation station setup. **A.** Electroporation station consists of microinjector (Eppendorf FemtoJet 4i, left), electroporator (TSS20 Intracel Ovodyne electroporator connected with EP21 current amplifier, right), dissecting stereomicroscope (middle) and left and right manual control micromanipulators placed on both sides of the stereomicroscope. The micromanipulators are mounted on magnetic base holders which are set on a 3 mm thick galvanized-iron base. **B** and **C.** Enlarged images of the microinjector and the electroporator respectively. **D.** Micropipette puller (Harvard Apparatus P-97 Flaming/Brown).

For setting up the electroporation station (**Fig. 1A**): (1) Obtain a commercially available galvanized iron sheet with approximate measurements of 80 cm length, 40 cm width, and 3 mm thickness, and then place the iron sheet in the middle of the electroporation station; (2) Place the stereomicroscope with its associated reflected light units in the middle of the iron sheet; (3) Place a manual micromanipulator (loaded on a mounting magnetic base) on each side of the stereomicroscope, one micromanipulator should hold the injection microcapillary holder and another should carry the electrodes’ holder; switch the magnetic controls of the mounting bases “on” to fix them firmly in place on the iron sheet; (4) Place the Eppendorf microinjector (**Fig. 1B**) on one side of the electroporation station and the electroporator (connected to the current amplifier) (**Fig. 1C**) on the other side; and (5) Connect the injection microcapillary to the microinjector using the provided tubing and the electrodes to the electroporator using the provided electric wires.

#### Micropipette pulling

**CRITICAL STEP:** Using the P97 micropipette puller (**Fig. 1D**), insert the microcapillary in position (see the P97 manual for illustration) and carry out a ramp test according to the manufacturer’s instructions (see P97 manual). This test determines at which temperature the glass microcapillary should start to melt. At the end of the ramp test, a heating value will be displayed on the puller’s screen. Use this heating value to set up a new microcapillary pulling program in which the heat value is set up within the range of +15 to **−**15 of the ramp test heating value to achieve the optimal microcapillary pulling.

The following program can be used as a guide to pull out the injection microcapillary:

Heat = ramp test heat value ± 15, pull: 40, velocity: 200, time: 50

**NOTE:** Pulling a very fine microcapillary tip is essential for carrying out neat and precise injections.

#### Electrodes assembly

**Figure 2.**
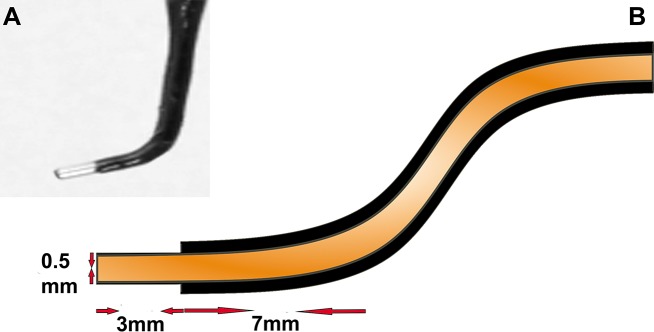
Electrodes assembly. **A.** Platinum wire assembled as L-shape. **B.** A diagram illustrates assembling the L-shape electrode as in (A) with measurements of different parts: 0.5 mm is the diameter of the electrode, 3 mm is the length of the bent part of the electrode, and 7 mm is the distance from the edge to the holding point. The current carrying part of the electrodes was made up by coating the electrodes with a nail varnish or any other non-current conducting coating material. The gap between the two electrodes can be set at 3–4 mm.

**CRITICAL STEP:** Assemble two lengths of Platinum wire in an L-shape (**Fig. 2A**) with one length serving as a negative (**−**ve) and another as a positive (+ve) electrode. The current carrying section of the electrodes should be 3 mm long (**Fig. 2B**) for precise tissue targeting and this can be made by coating the remaining bent part of the electrodes with a nail varnish thus allowing a small window to permit the passing of current between the −ve and +ve electrodes. The distance from the edge to the holding point should be 7 mm long. Solder the assembled electrodes to a black (−ve electrode) and red (+ve electrode) electric wires. Check the conductivity and resistance after soldering. Mount both the −ve and +ve electrodes on the electrodes’ holder by which the gap between the electrodes is approximately 3–4 mm. Mount the electrodes’ holder onto the left-handed side micromanipulator and fix it using its magnet switch on the left-hand side of the stereomicroscope.

**NOTE:** (1) Coating of the electrodes prevents damage to the embryos and increases the precision of the tissue targeting; and (2) Platinum electrodes with Platinum coating can be bought ready-made (BEX, fixed Platinum needle electrode, LF613 series, LF611P7-3).

#### DNA preparation

**Figure 3.**
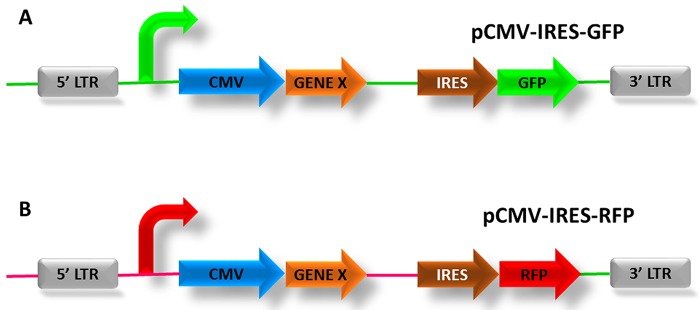
Control expression vectors for electroporation. **A.** pCMV-IRES-GFP construct (Cytomegalovirus- Internal Ribosome Entry Site-Green Fluorescence Protein) in which the gene of interest (Gene X) can be sub-cloned into the multiple cloning site. The β-actin promoter with the CMV enhancer drives the expression of the sub-cloned gene and an IRES, and a nuclear variant of GFP allows the two proteins to be translated separately. **B.** pCMV-IRES-RFP construct is the same construct as in (A) but RFP (red) is expressed instead of GFP.

Expression vectors carrying GFP or RFP such as pCMV-IRES-GFP (**Fig. 3A**) or pCMV-IRES-RFP (**Fig. 3B**) can serve as a control electroporated DNA. Extract DNA using Qiagen EndoFree Plasmid Maxi kit according to the manufacturer’s instructions. Carefully measure the DNA concentration using NanoDrop based on which DNA can be further concentrated by EtOH precipitation to obtain a concentration ranging from 1 mg/ml to a maximum of 3 mg/ml. Wash DNA pellet twice with 70% EtOH to eliminate any salts interference which could lower the electroporation efficiency. DNA at 1–2 mg/ml should give good fluorescence intensity of GFP or RFP after electroporation. A DNA concentration above 3 mg/ml will be too viscous and difficult to inject due to clogging of the microcapillary.

**HINTS:** (1) In case of the expression vectors which do not have GFP (such as pCDNA3 vector, Thermo Fisher cat. # V79020), mix with 2–3 µl of 1 mg/ml IRES-GFP vector to enable tracing the injected DNA (for example: add 15 µl of 2 mg/ml pCDNA3 + 3 µl 1 mg/ml pCMV-IRES-GFP); and (2) Centrifuge DNA mix at 12000 rpm for 2 min at room temperature before using it for microinjection.

#### Morpholino preparation

Dissolve control morpholino (CMO) according to the manufacturer’s instructions in double distilled autoclaved water at a concentration of 300 mM.

**CRITICAL STEP:** MO should be heated up to 60°C for 5 min to denature before injection and then keep on ice during injection. Store after injection at 4°C.

**HINT:** If fluorescently-labelled MO (yellow/greenish in color) is used, there is no need to mix it with Fast Green since it will be clearly visible during injection.

**NOTE:** (1) Fluorescently-labelled CMO sequence should not target any other gene’s sequence; and (2) It can be detected after 4 h of electroporation by tracing the green fluorescence expression using Alexa Fluor 488 ηm GFP filter.

#### Microcapillary assembly

Fill in the microcapillary with DNA/Fast Green mix or fluorescent CMO using Eppendorf microloader fine tip and then mount the microcapillary onto the microcapillary holder. Mount the holder on the right-handed-side micromanipulator and fix it on the right-hand side of the stereomicroscope. Under the stereomicroscope, snip off the tip of the microcapillary using the Watchmaker’s forceps No. 5. Test the flow of the DNA or MO out of the microcapillary by continuously pressing the inject button of the Eppendorf FemtoJet microinjector until a bolus of the DNA or MO starts to come out of the needle’s tip. If this does not happen, then carefully snip off the tip of the microcapillary again until the DNA or MO bolus can be seen coming out of the needle’s tip. Try to keep the needle’s tip as fine as possible.

## PROCEDURE

**Figure 4.**
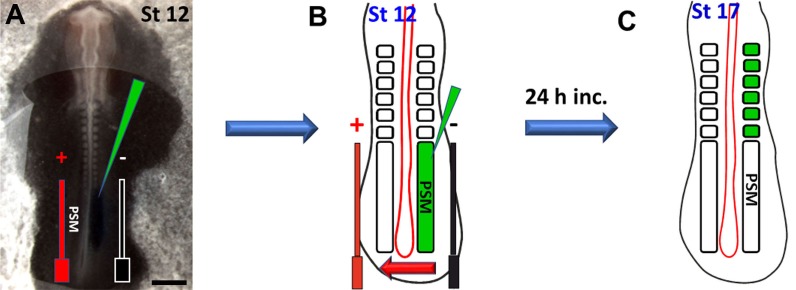
Electroporation into the chick presegmented mesoderm to target the epithelial somites. **A.** Embryo at stage HH12 showing injection into the PSM, the green triangle represents the injection microcapillary, black and red bars indicate the −ve and +ve electrodes respectively. **B.** Schematic diagram of the caudal part of the embryo in (A) illustrating the injection of the fluorescently-labelled CMO into the PSM after which the embryo was re-incubated for 24 h. The red arrow indicates the direction of the current. **C.** Schematic diagram for the embryo in (A) illustrating the electroporated PSM after segmentation showing GFP expression in the epithelial somites. Scale bar: 0.5 mm in (A).

**Figure 5.**
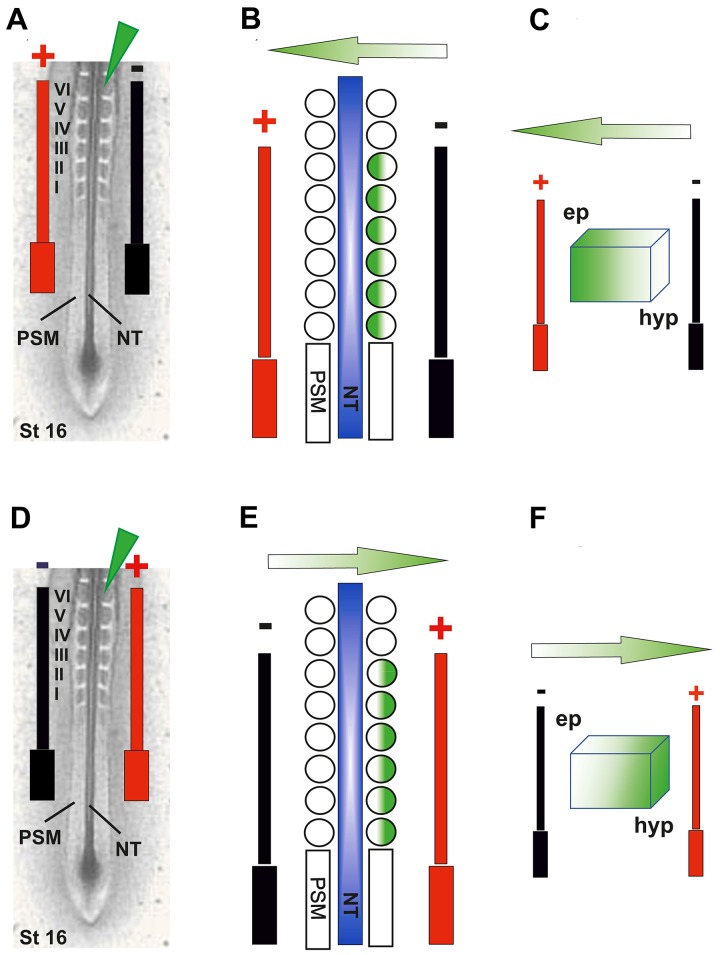
Electroporation of the epithelial somites. **A-C.** Schematic diagrams represent injection of the GFP into the epaxial domain of the ES at stage HH16, black and red bars indicate the position of the −ve and +ve electrodes respectively. **D-F.** Schematic diagrams represent electroporation of the hypaxial domain of the ES in which similar injection and electroporation into the ES as in (A-C) can be carried out but the current is reversed by switching the position of the −ve and +ve electrodes. Green arrows in (B, C) and (E, F) indicate the direction of the current when electroporating either the epaxial or hypaxial domains respectively.

1. Embryo manipulation
  ***1.1.*** Take an egg out of the incubator, and then wipe out the blunt end (air space side) with 70% EtOH, place the egg under the stereomicroscope with its blunt end facing up, and stick a small strip of tape on the eggshell blunt end.
  ***1.2.*** Using a pair of small dissecting scissors, cut through the tape and the underneath eggshell to make a circle of 1–1.5 cm diameter egg window.
    **HINT:** Care should be taken while making the egg window not to damage the underneath embryo. This can be achieved by keeping the dissecting scissors in a horizontal position while cutting through the eggshell.
  ***1.3.*** Add 2–3 drops of PBS/Penicillin-Streptomycin (10 μl/ml) on top of the outer vitelline membrane and then gently pierce the membrane using the Watchmaker’s forceps No. 5. PBS should infiltrate through the membrane which then swells and can be easily ruptured and removed by the forceps. The embryo should be immediately exposed after the membrane removal.
    **HINT:** (1) Contamination could be a major problem and hence Penicillin-Streptomycin should be added to the PBS in all steps of the embryo manipulations; and (2) Dissecting tools should be sterilized either with 70% EtOH or by autoclaving before use.
  ***1.4.*** Inject Indian ink buffer using 1 ml syringe with 26-gauge needle underneath the blastoderm disc to visualize the embryo.
  ***1.5.*** Carefully remove the inner vitelline membrane on top of the embryonic disc at the site where the tissue of interest to be electroporated using a very fine Tungsten needle.
2. Neural tube electroporation and optimization
  ***2.1.*** At stage HH16 embryos, make a small egg window as described in step 1, remove the outer and inner vitelline membranes on top of the NT, and then manoeuvre and gently insert the microcapillary containing DNA mix (1–2 mg/ml IRES-GFP/Fast Green) into the NT lumen.
  ***2.2.*** Fill in the NT with the DNA, gently take out the microcapillary, and then carefully place the electrodes to sandwich the NT. Add 2–3 drops of PBS/Penicillin-Streptomycin and electroporate the DNA. Add immediately 2–3 drops of PBS to cool down the electroporation site and then gently elevate the electrodes using the micromanipulator.
  ***2.3.*** Test a range of volts, *e.g.*, 15, 20, 25, 30 and 35 volts and a number of pulses, *e.g.*, 3, 4, 5, and 6 pulses.
    **NOTE:** Achieving optimal electroporation conditions will depend on several important factors, among these: (1) type of the electrodes, *e.g.*, platinum or tungsten; (2) electrodes’ diameter (usually between 3 to 5 mm); (3) the gap between the −ve and +ve electrodes (usually between 3–5 mm); (4) type of tissue to be electroporated; and (5) type of the electroporator.
    **NOTE:** It is important to take in consideration that careful assembly of the electrodes combined with the critical optimization of the electroporation parameters are essential in producing a highly efficient electroporation.
  ***2.4.*** Aspirate some of the egg albumen using 10 ml syringe with 18-gauge needle until the embryo is slightly brought down. Seal the egg with tape and re-incubate until the desired embryo stage is reached. Embryos can be checked at any time during incubation.
    **NOTE:** (1) Once the embryos are electroporated and re-incubated, rotating the incubator’s shelves should be stopped to avoid adhesion of the embryos to the sealing tape; and (2) The egg window should be well-taped to avoid gaps which could lead to leakage of hot air into the embryos which will affect their survival.
  ***2.5.*** Check GFP signals after 4 to 24 h of DNA electroporation. IRES-GFP can be detected by Alexa Fluor 488 ηm filter and IRES-RFP by Alexa Fluor 565 ηm filter. After incubation is complete, dissect out the electroporated embryos.
  ***2.6.*** Assess the electroporation efficiency based on the following parameters (see also **Table S1**).
    **2.6.1.** The overall embryo survival: should be at least 90%.
    **2.6.2.** The embryo morphology: at least 90% of normal development should be achieved.
    **2.6.3.** The intensity of the GFP signals: this can be classified into four levels as follows: (1) Absent (level 0): No signals can be detected; (2) Weak (level 1): Few or scattered GFP signals can be detected; (3) Moderate (level 2): Less than or 50% of the electroporated tissue shows strong GFP signals; and (4) Strong to very strong (level 3): Very bright GFP signal intensity can be easily detected in majority of the electroporated tissue.
    **2.6.4.** Electroporation can be considered as successful when it results in a high survival rate, and normal morphology but associated with strong or very strong GFP fluorescence intensity.
    **HINTS:** (1) If the electroporation is producing kinky embryos, then this suggests that the electroporation conditions are not properly optimized. The voltage (v) could be lowered as well as the number of pulses until normal embryo development is achieved; and (2) Take images before fixation since PFA fixative could cause auto-fluorescence and thus gives false signals.
3. Presegmented mesoderm electroporation
  ***3.1.*** Manipulate stage HH12 embryos as described in step 1, and then manoeuvre the microcapillary containing DNA mix or MO to approach the PSM. Carefully insert the microcapillary fine tip into the rostral domain of the PSM. Inject the molecule to be electroporated which should spread in a rostrocaudal direction of the PSM until it is filled (**Fig. 4A** and **4B**).
  ***3.2.*** Place the electrodes gently to sandwich the PSM and then add 2–3 drops of PBS/Penicillin-Streptomycin before DNA is electroporated. Apply the optimized parameters obtained from the NT electroporation to electroporate the PSM (**Fig. 4B** and **4C**).
  ***3.3.*** Follow steps 2.4–2.6.
4. Epithelial somites electroporation
  ***4.1.*** At stage HH16, manipulate the embryos as in step 1, manoeuvre the microcapillary to approach the first ES and gently insert the tip to pierce the somite epithelium until it reaches the somitocoel and then inject the DNA. Similarly, inject the rest of the ESs to be electroporated. Gently place the electrodes to sandwich the somites, add 2–3 drops of PBS and electroporate the epaxial domain (**Fig. 5A-5C**). If the hypaxial somite domain is to be electroporated, then similarly inject the somite but reverse the current as shown in **Fig. 5D-5F**.
    **HINT:** Keep the tip of the microcapillary as fine as possible during the injection which not only helps in reducing any possible damage to the somite but will also minimize any leakage of the injected DNA.
  ***4.2.*** After incubation is completed, dissect out the electroporated embryos and then assess the embryos’ survival, normal morphology and GFP signals intensity as described in step 2.6.
5. Embryo fixation, *in situ* hybridization and immunostaining
  ***5.1.*** Using a pair of dissecting scissors, carefully remove the electroporated embryos in a Petri-dish containing DEPC-PBS. Carefully transfer the embryos using a 5 ml plastic Pasteur pipette with its tip cut off.
  ***5.2.*** Remove the embryonic membranes using Watchmaker’s forceps No. 5 before capturing images.
  ***5.3.*** Fix the embryos overnight at 4°C in 4% PFA/DEPC-PBS with rocking using a tube roller.
  ***5.4.*** Wash embryos after fixation in DEPC-PBS then analyze the target gene(s) by either ISH protocol as reported in Geisha [16] and/or immunostaining as described by Abu-Elmagd *et al*. 2001 [18]. Double ISH can be carried out as described by Abu-Elmagd *et al*. 2010 [19].
  ***5.5.*** mRNA probe synthesis for ISH can be carried out as described in Geisha protocols [16].

## ANTICIPATED RESULTS AND DISCUSSION

**Figure 6.**
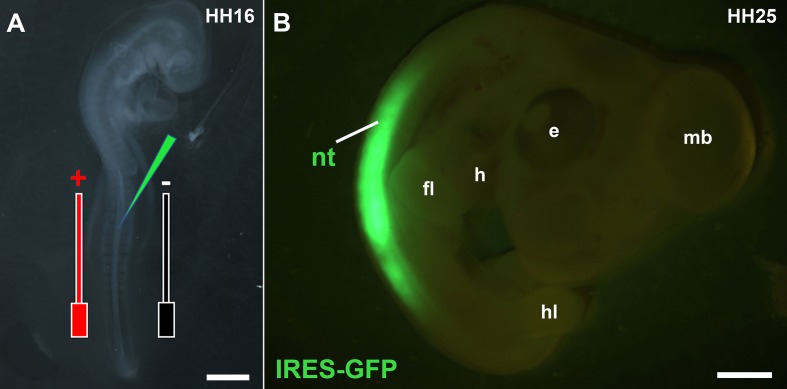
Electroporation of the chick neural tube. **A.** Stage HH16 chick embryo showing injection of the IRES-GFP (2 mg/ml) into the NT. Black and red bars indicate the position of the –ve and +ve electrodes respectively. **B.** Electroporated embryo at HH16 in the NT and incubated till stage HH25 showing strong GFP signals in the NT, note the overall normal morphology of the embryo. e: eye, h: heart, fl: fore-limb, hl: hind-limb, mb: mid-brain. Scale bars: 0.5 mm in (A), 2 mm in (B).

The current protocol consists of two stages (see the graphical abstract): In the first stage, the NT of HH16 chick embryo (**Fig. 6A**) was used as a tool to optimize the electroporation conditions. To achieve this, we initially tested a number of voltages (15, 20, 25, 30, and 35). Each voltage was combined with 5 pulses (p), 30 ms pulse space (ms/ps) and 100 ms pulse width (ms/pw). After each experiment, the efficiency of the electroporation was assessed as described in step 2.6 by which strong to highly strong GFP or RFP signals (level 3) constituted efficient or successful electroporation. For each voltage we tested, 50 embryos were electroporated (total *n* = 250 embryos for all voltage trials). The results revealed that only 25 v combined with 5 p, 30 ms/ps and 100 ms/pw led to achieving level 3 of GFP expression. After this initial electroporation parameters testing, we applied 25 v, 5 p, 30 ms/ps, and 100 ms/pw to electroporate the NT in three separate experiments to ensure reproducibility (total *n* = 147 embryos). Each experiment resulted in a survival rate and normal development of at least 96% of the electroporated embryos (total *n* = 141). This was combined with level 3 of GFP signals intensity in 95% of the embryos (*n* = 139) (**Fig. 6B**). According to the criteria established in this protocol, we considered these results to represent efficient electroporation.

Number of pulses was another important parameter to test during the electroporation optimization stage. A varying number of pulses (3, 4, 5, and 6) in a combination of 25 v, 30 ms/ps, and 100 ms/pw were applied to electroporate the NT. Three (*n* = 46 embryos) and four (*n* = 41 embryos) pulses resulted in either absence (level 0) or weak (level 1) GFP signals. Five pulses were tested as mentioned above. Six pulses (*n* = 43) resulted in very strong GFP signals but this was associated with either a low survival rate and/or kinky embryos. At the end of this optimization stage, we concluded that 25 v, 5 p, 30 ms/ps, and 100 ms/pw were the best electroporation parameters to apply in the electroporation of the PSM and ES.

**Figure 7.**
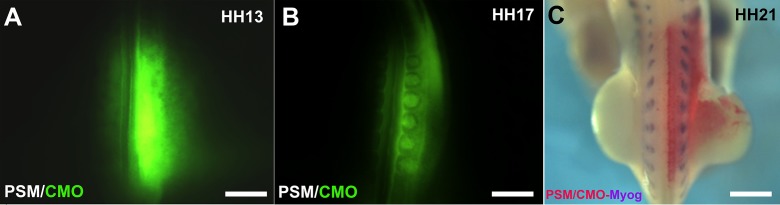
Control morpholino electroporation in the presegmented mesoderm. **A.** Embryo electroporated with CMO in the PSM with the green fluorescence signals detected after 4 h of electroporation. **B.** The embryo in (A) with the green fluorescence signals detected in the ES after 24 h of incubation. **C.** Embryo electroporated in the PSM at stage HH12 with CMO incubated for 3 d and then subjected to the ISH to detect the CMO (using GFP probe and detected by Fast red) and Myog (detected in blue by NBT/BCIP). Injected and electroporated somites showed normal Myog endogenous expression as well as the non-injected and electroporated control side. Scale bars: 0.5 mm in (A), 0.4 mm in (B), 0.3 mm in (C).

In stage 2, we applied the optimal NT electroporation conditions to electroporate MO into the PSM. This showed efficient electroporation results similar to those produced by NT electroporation using 25 v, 5 p, 30 ms/ps, and 100 ms/pw program. This was indicated by strong GFP signals (level 3) after 3–4 h of electroporation (**Fig. 7A**) (total *n* = 121, 94%, three experiments). Green fluorescent MO was easily detectable later in the ES (**Fig. 7B**). PSM electroporated embryos also showed normal morphology in at least 93% (total *n* = 112). In addition, the effects of the electroporation on the endogenous gene expression were checked by ISH which showed normal Myog expression (**Fig. 7C** as an example, 100%). The non-injected but electroporated left side of the embryo served as the internal control.

**Figure 8.**
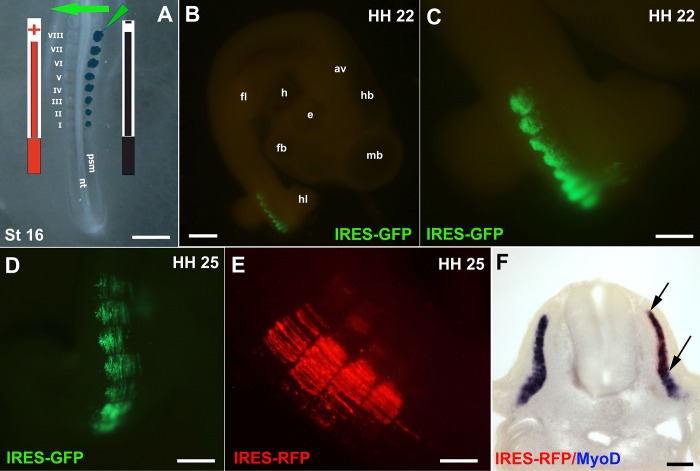
Electroporation of the epithelial somites. **A.** Injection and electroporation of pCMV-IRES-GFP into the ES at stage HH16. Light green triangle represents the injection microcapillary and dark green dots represent where DNA was injected. Black and red bars indicate the position of the −ve and +ve electrodes respectively in relation to the ES. **B.** Example of an embryo electroporated with IRES-GFP at stage HH16 then incubated for 24 h, strong GFP fluorescence can be seen in somites. **C.** Enlarged electroporated somites of the embryo in (B). **D.** Embryo electroporated with IRES-GFP at stage HH16 and incubated for 72 h, strong GFP fluorescence signals can be seen in the somites. **E.** Embryo electroporated with IRES-RFP at stage HH16 and incubated for 72 h, RFP strong fluorescence signals can be seen in the somites. **F.** Transverse section of the embryo in (E) after processing for double ISH showing RFP (red) expression in the transfected somite domain (arrows) and MyoD expression (blue) in both injected (right) and non-injected control sides (left). GFP in (B-D) was detected by Alexa Fluor 488 ηm and RFP in (E) by Alexa Fluor 565 ηm fluorescence filters. av: auditory vesicle; e: eye; ES: epithelial somites; fb: fore-brain; fl: fore-limb; h: heart; hb: hind-brain; hl: hind limb; mb: mid-brain; nt: neural tube; psm: presegmented mesoderm. Scale bars: 0.5 mm in (A and D), 1.5 mm in (B), 0.4 mm (C), 0.3 mm in (E) and 0.1 mm in (F).

The electroporation program (25 v, 5 p, 30 ms/ps, and 100 ms/pw) applied to the NT and PSM was used to electroporate the epaxial domain of the ES (total *n* = 152, three experiments) (**Fig. 8A**). These conditions showed reproducible and efficient results indicated by strong (level 3) GFP signals (**Fig. 8B** and **8C**) (94%, total *n* = 142). When the electroporated embryos were incubated for up to 3 d, they showed strong GFP expression with no signs of abnormal development (**Fig. 8D**). Similarly, efficient electroporation results were obtained when IRES-RFP was electroporated (**Fig. 8E**) (total *n* = 88, 92%, three experiments). The effects of IRES-RFP electroporation on the endogenous MyoD expression was checked by ISH which showed normal expression pattern in both electroporated and injected somites as well as the electroporated non-injected control side (**Fig. 8F**).

*In vivo* electroporation is based mainly on applying current to the tissue of interest at a low voltage to cause a temporary perforation of the cell membrane to allow uptake of exogenous molecules. Such method proved to be a powerful approach for gene transfer and functional studies. Our results showed that the NT is highly suitable for electroporation due to its inherent structural advantages, including a narrow lumen that can retain the DNA after injection, as well as a dorsoventral structural depth—making it highly receptive to the current. These advantages allowed us to utilize this robust system to easily optimize the electroporation parameters and to apply these parameters to electroporate the PSM and ES. However, additional parameters including proper electrodes’ assembly, microcapillary assembly, concentration of the injected molecule(s), and purity and quality of the injected DNA should be all considered to achieve efficient electroporation.

The current approach to optimize the electroporation parameters using the NT is not limited to electroporating the PSM and ES. Our previous work has showed that the same approach for optimizing the electroporation conditions can be applied to electroporate other tissues such as the epibranchial placodes [18] and surface ectoderm [20]. Furthermore, we previously used the same approach to successfully electroporate more than one molecule such as two morpholinos, and a mix of double strand DNA and MO [13]. In conclusion, the current protocol may be of use in the electroporation of embryonic tissues which are considered to be “difficult to electroporate” such as the somites, optic and auditory vesicles, epibranchial placodes, and heart.

## TROUBLESHOOTING

Potential problems that could arise during the electroporation protocol and suggestions to troubleshoot them are listed below in **Table 1**.

## Supplementary Material

Supplementary information **Table S1**. Electroporation efficiency assessment sheet of embryo survival, normal/abnormal development and GFP expression intensity in relation to the electroporation parameters.
Supplementary information of this article can be found online at
http://www.jbmethods.org/jbm/rt/suppFiles/253.

## Figures and Tables

**Table 1.** Troubleshooting.

Step	Problem	Cause	Suggestions
Egg incubation	Not obtaining a good number of normal embryos to operate or a developmental delay	Fertilized chicken eggs are of low quality	Consult the eggs supplier Closely monitor the setting of the incubator’s temperature and humidity Continuously rock the eggs during incubation
Electrodes assembly and/or NT, PSM and ES electroporation	Electrodes burn the electroporation site	Electrodes are incorrectly assembled Unoptimized electroporation conditions	Use Platinum electrodes with a smaller diameter (*e.g.*, 3 mm) Try Tungsten electrodes (LF614T, BEX Co., Ltd.) or stainless-steel electrodes (BEX Co., Ltd., LF614S) to reduce damage to the embryo Optimize electroporation conditions by reducing the voltage and/or number of pulses Make sure that 2–3 drops of PBS are added before electroporation
Micropipette pulling	Neels’s puller does not pull a very fine microcapillary	Pulling program is not properly optimized	Run a ramp test and obtain a heating value guide Increase the heating value and reduce the pulling force
Microcapillary assembly and/or DNA/morpholino preparation	Injected DNA or MO leaks out from the injected tissue	Needle does not have a very fine-tip and not properly pulled Low concentration of the injected molecule	Pull very fine microcapillary Increase DNA concentration
Step 2.6	Low survival rate after electroporation	Possible contamination problem Unoptimized electroporation conditions	Make sure that Penicillin/Streptomycin antibiotic is added to an autoclaved PBS buffer Decrease the voltage Decrease the number of pulses
Step 2.6	Electroporation produces kinky or malformed embryos	Unoptimized electroporation conditions	Try different voltages Try different pulses Try different combinations of voltages and number of pulses
Step 2.6	GFP expression after electroporation is weak or moderate	Fluorescence microscope is not properly aligned Poor DNA quality and/or low DNA concentration Unoptimized electroporation conditions	Check the stereo fluorescence microscope setting, ensure that its fluorescence beam is properly aligned Make sure you use high quality DNA, check DNA/RNA ratio Make sure that DNA concentration exceeds 1 mg/ml For MO, make sure you heat it up to 60°C and then keep on ice for 10 min before electroporation Reduce the gap between the two electrodes to 3 or 4 mm Carefully increase the voltage Carefully increase the number of pulses
Anticipated results (for the electroporated embryos after ISH)	Gene expression of a target gene (detected by ISH) is missing in embryos electroporated with a control IRES-GFP either in the injected or non-injected side	Cellular damage induced by unoptimized electroporation conditions	Decrease the voltage Decrease the number of pulses
